# EFFECT OF HEARING INTERVENTION ON SOCIAL FUNCTION, DEPRESSION, AND HEALTH-RELATED QUALITY OF LIFE IN OLDER ADULTS

**DOI:** 10.1093/geroni/igad104.0299

**Published:** 2023-12-21

**Authors:** Alison Huang

**Affiliations:** Johns Hopkins Bloomberg School of Public Health, Baltimore, Maryland, United States

## Abstract

Prior observational studies show associations between hearing loss and worse social and mental health among older adults. Whether hearing loss treatment is an effective intervention is unknown. The Aging and Cognitive Health Evaluation in Elders Study (ACHIEVE) randomized controlled trial (n=977) tests the effect of a best-practices hearing intervention vs. successful aging health education control intervention on pre-specified exploratory outcomes including social isolation (Cohen Social Network Index), loneliness (UCLA Loneliness Scale), depressive symptomology (11-item Center for Epidemiologic Studies Depression scale), and health-related quality of life (RAND-36 Health Survey). Generalized linear mixed effects models test if 3-year change in social and mental health outcomes differ by intervention assignment. Participants have untreated hearing loss and are 70-84 years, 88% White, 54% female, and 53% had a Bachelor’s degree or higher. At baseline, prevalence of loneliness was higher among those with moderate or greater (69%) compared to mild (64%) hearing loss. Physical health-related quality of life was lower among those with moderate or greater (mean[standard deviation(SD)]: 44[10]) compared to mild (46[9]) hearing loss. Prevalence of depression (4%), social network characteristics (e.g., mean(SD) social network size: 22[11] members), and mental health-related quality of life (mean(SD): 56[7]) were similar by hearing loss severity. This presentation focuses on hearing intervention effects and results will be available by the time of the conference (data collection ends early 2023). Existing interventions for improving social and mental health are limited in effect size and scalability. Hearing treatment may be an effective intervention with potential for greater widespread implementation.

